# Spatial patterns of hypolithic cyanobacterial diversity in Northern Australia

**DOI:** 10.1002/ece3.3248

**Published:** 2017-07-31

**Authors:** Keith Christian, Mirjam Kaestli, Karen Gibb

**Affiliations:** ^1^ Research Institute for the Environment and Livelihoods Charles Darwin University Darwin Northern Territory Australia

**Keywords:** biogeography, colonization, cyanobacteria, hypolithic, soil microbiota, spatial patterns

## Abstract

Photosynthetic microbial communities under translucent rocks (hypolithic) are found in many arid regions. At the global scale, there has been little intercontinental gene flow, and at a local scale, microbial composition is related to fine‐scale features of the rocks and their environment. Few studies have investigated patterns of hypolithic community composition at intermediate distances. We examined hypolithic cyanobacterial diversity in semi‐arid Australia along a 10‐km transect by sampling six rocks from four adjacent 1 m^2^ quadrats (“distance zero”) and from additional quadrats at 10, 100, 1,000, and 10,000 m to test the hypothesis that diversity would increase with the number of rocks sampled and distance. A total of 3,108 cyanobacterial operational taxonomic units (OTUs) were detected. Most were neither widespread nor abundant. The few that were widespread tended to be abundant. There was no difference in the community composition between the four sites at distance zero, but the samples 10 m away were significantly different, as were those at all other distances compared to distance zero. Many additional OTUs were recorded with increasing distance up to 100 m. These patterns of distribution are consistent with a colonization model involving dispersal from rock to rock. Our results indicate that distance was a significant factor that can be confounded by interrock differences. Most diversity was represented in the first 100 m of the transect, with an additional 1.5% of the total diversity added by the sample at 1 km, but only 0.2% added with the addition of the 10‐km site.

## 1 INTRODUCTION

Photosynthetic microbial communities inhabiting the underside of translucent rocks (hypolithic) are found in many arid (Pointing, 2016; Warren‐Rhodes et al., 2006) and semiarid (Tracy et al., 2010) regions, including both hot and cold deserts (Chan et al., 2012; Pointing & Belnap, 2012; Smith et al., 2000). These communities are dominated by cyanobacteria (Valverde et al., 2015), although other components such as fungi and mosses may be present in some environments (Cowan et al., 2011; Makhalanyane, Valverde, Birkeland, et al., 2013). Hypolithic cyanobacteria have the capacity to fix carbon and nitrogen, and they constitute a substantial component of biomass in some arid regions (Chan et al., 2012; Heckman, Anderson, & Wait, 2006; Makhalanyane, Valverde, Birkeland, et al., 2013; Pointing & Belnap, 2012). These characteristics suggest that they have important ecosystem functions in arid habitats, although landscape scale studies remain to be carried out (Pointing, 2016).

Physical factors that limit photosynthetic activity, and hence distribution of hypolithic cyanobacteria, include solar radiation (Cowan et al., 2011; Tracy et al., 2010), temperature (Schlesinger et al., 2003; Tracy et al., 2010; Warren‐Rhodes et al., 2007), water from rain (Tracy et al., 2010; Warren‐Rhodes et al., 2006), snowmelt (Warren‐Rhodes et al., 2007), and fog (Azúa‐Bustos et al., 2011; Warren‐Rhodes et al., 2013). These environmental factors can influence both the extent to which translucent rocks support cyanobacterial communities and the diversity of the communities (Cowan et al., 2011; Heckman et al., 2006; Warren‐Rhodes et al., 2006, 2013).

Given the ancient origins of cyanobacteria and the uncertainties around the colonization mechanisms between suitable rocks (Pointing, 2016; Warren‐Rhodes et al., 2007), the diversity of hypolithic cyanobacterial communities is interesting at the global, regional, and local scales. At the global scale, analyses of community composition indicate that stochastic processes have been important in shaping patterns of community composition (Caruso et al., 2011). In a study of *Chroococcidiopsis* spp. (Bahl et al., 2011), the authors concluded that “global distribution of desert cyanobacteria has not resulted from widespread contemporary dispersal but is an ancient evolutionary legacy.” At the landscape scale (~10–100 km), species richness and diversity increase with increasing water availability (Pointing et al., 2007; Stomeo et al., 2013; Warren‐Rhodes et al., 2006, 2007).

At a local scale, the abundance of colonized rocks is related to fine‐scale features of the rocks, the soil, and topographic properties that influence water availability (Warren‐Rhodes et al., 2007). Wong et al. (2010) found that the community composition was similar under rocks sampled within an area of 100 m^2^, but few studies have investigated patterns of hypolithic cyanobacterial community composition at distances small enough that climatic factors do not vary but great enough that immediate rock to rock dispersal is unlikely. Patterns of microbial distributions over space have been studied by examining the relative importance of distance (implying dispersal limitations) and environmental selection (correlations with microhabitat characteristics) (Lacap, Lau, & Pointing, 2011; Martiny et al., 2011). Here, we report patterns of cyanobacterial community diversity across a 10‐km transect at a semi‐arid site in northern Australia. The transect was across a homogeneous landscape with no differences in altitude or proximity to waterways, and no apparent differences with respect to soil type, vegetation structure, or topography. Thus, although no soil measurements were taken, the microhabitat characteristics did not vary in any apparent way, allowing an examination of microbial diversity primarily as a function of distance.

In a previous study at this site (Tracy et al., 2010), nine rocks were sampled opportunistically, with the main goals of comparing the communities with those from other deserts of the world and describing the hypolithic communities under different rock types (quartz crystals, quartz matrix (small crystals resulting in a milky appearance), agate, and prehnite). The hypolithic communities were diverse under all the rock types, and the high diversity from such a small sample of rocks suggested that a larger sample might reveal many more species. Here, we test the hypothesis that a systematic sampling regime of a larger sample of quartz rocks along a 10‐km transect would reveal increasing diversity with increasing sample size and distance from an initial point. We also address questions related to the number of rocks that needed to be sampled to determine a representative list of the cyanobacterial community composition, a richness estimate of the cyanobacterial operational taxonomic units (OTUs) per rock and how this varies with location and rock size, and cyanobacterial diversity as a function of rock characteristics (size, quartz crystal, or quartz matrix).

## 2 MATERIALS AND METHODS

### 2.1 Study site and sampling scheme

The study site was ~10 km south of Kalkarindji, NT, Australia (Tracy et al., 2010). Vegetation at the site is dominated by spinifex grass (*Triodia* sp.), with sparse shrubs and eucalyptus trees. The annual mean rainfall is ~690 mm (Tracy et al., 2010).

**Figure 1:**
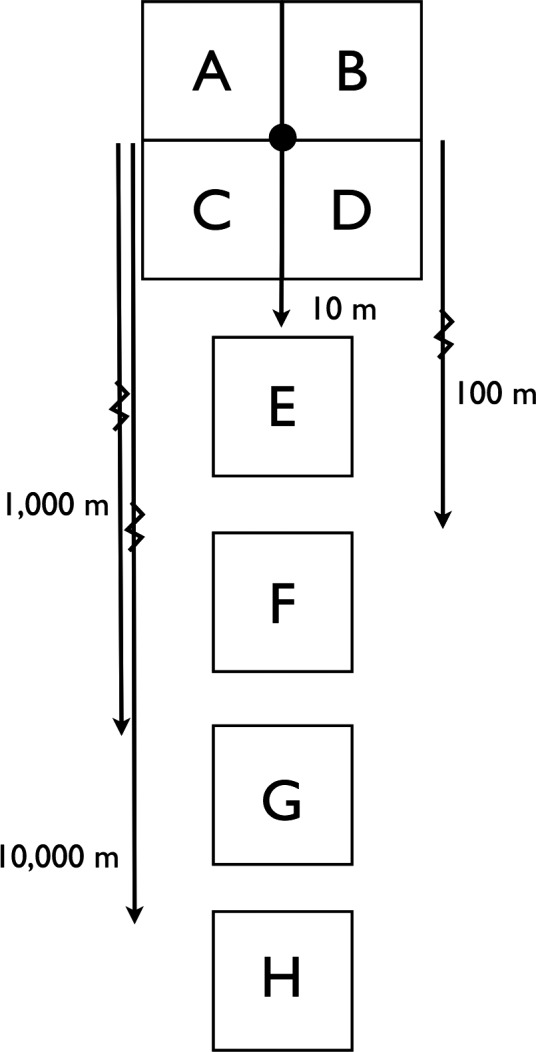
The sampling scheme in which each square represents a 1 m^2^ quadrat from which six quartz rocks with cyanobacteria were collected. The common midpoint corner of quadrats A, B, C, and D was defined as distance zero, and quadrats E, F, G, and H were located10, 100, 1,000, and 10,000 m south of that point

To describe the cyanobacterial diversity across a 10‐km transect, we sampled one site intensively to determine the effects of sample size from a given location. This location consisted of four adjacent 1 m^2^ quadrats labeled A, B, C, and D, and the intersection of these quadrats was designated as “distance zero.” Four additional quadrats (E, F, G, and H) were located 10, 100, 1,000, and 10,000 m south of this point. The sampling scheme is illustrated in Figure 1, and it allows an evaluation of species richness and cyanobacterial diversity at the level of the individual rock, short distances, and longer distances. Six quartz rocks were collected from each quadrat in August 2014. In addition to its location of origin, we measured the following physical characteristics of each rock: mass (g), length (mm), width (mm), depth (mm), and whether the quartz consisted of small crystals (matrix) or larger crystals (crystals).

### 2.2 Biofilm DNA extraction

Rocks were kept at room temperature until a scalpel blade was used to lift biofilm off each rock, and DNA was extracted from 0.2 g using the MoBio PowerBiofilm^®^ Powerlyser DNA Isolation Kit (MoBio Laboratories, CA, USA) following the manufacturer's instructions.

## 3 16S rDNA TARGET SEQUENCING

Two μg of DNA was sent to the sequencing provider mrdnalab (www.mrdnalab.com, Shallowater, TX, USA) for amplification using the primers CYA359F (5′‐GGGGAATYTTCCGCAATGGG) and CYA809R (5′‐GCTTCGGCACGGCTCGGGTCGATA) targeting the 16s rRNA genes of cyanobacteria (Baker, Entsch, & McKay, 2003; Miller et al., 2005). The forward primers contained sample specific 8‐nucleotide barcodes. The HotStarTaq Plus Master Mix Kit (Qiagen, USA) was used with the following conditions: 94°C 3 min, 28 cycles of 94°C 30 s, 53°C 40 s, 72°C 1 min, final elongation step at 72°C for 5 min. Following PCR, all amplicon products from different samples were mixed in equal concentrations and purified using Agencourt Ampure beads (Agencourt Bioscience Corporation, MA, USA). Samples were sequenced utilizing Roche 454 FLX titanium instruments and reagents and following manufacturer's guidelines.

### 3.1 Processing of sequencing data

Sequence data were processed using a proprietary analysis pipeline (www.mrdnalab.com, MR DNA, Shallowater, TX). Sequences were depleted of barcodes, primers, and short sequences <200 bp were removed as well as sequences with ambiguous base calls and homopolymer runs exceeding 6nt. Chimeras were also removed. OTUs were defined after removal of singleton sequences, clustering at 3% divergence (97% similarity) (Capone et al., 2011; Dowd et al., 2008; Edgar, 2010; Swanson et al., 2011). OTUs were taxonomically classified using BLASTn against a curated GreenGenes database (DeSantis et al., 2006).

### 3.2 Data analysis

Data were analyzed in R (version 3.2.2.) using the packages phyloseq in Bioconductor (Callahan et al., 2016) and corrplot; in Primer‐7 (Primer‐E, Plymouth, UK), Estimate‐S (version 1.9.0, Colwell et al., 2012), Stata‐14 IC (STATA Corp, Texas, USA), and Cytoscape (version 3.4.0, www.cytoscape.org).

The number of sequences was compared between samples, and samples with outlying low number of sequences were excluded from the analysis. OTUs which occurred in only one sample were excluded. Due to the choice of primers targeting the 16S rRNA genes of cyanobacteria, OTUs whose taxonomic classification differed from cyanobacteria were excluded.

The number of sequences per sample was compared to the number of OTUs and three different OTU normalizing or standardizing techniques were tested, namely (1) standardizing by relative abundance, (2) rarefying to the lowest common number of sequences after exclusion of samples as per above, or (3) applying a variance stabilizing normalization implemented in deseq2 in phyloseq. OTU data were square root transformed to down‐weight highly abundant OTUs for (1) and (2) and a constant (2.0692) added to all values of (3) to transform negative values back to positive. A Bray–Curtis similarity matrix was calculated in Primer‐7 on the transformed OTU data. A second stage analysis was conducted in Primer‐7 comparing the distance matrices of (1), (2), and (3) using nonparametric Spearman's correlations. PERMANOVA analysis described below was performed on OTU data normalized by all three techniques and results were compared.

To test for differences in the cyanobacterial community between distances, a PERMANOVA analysis was conducted in Primer‐7 on the Bray–Curtis distance matrix of the standardized and square root transformed OTU data with distance as a categorical fixed factor. A heat‐map triangle of the average Bray–Curtis similarities between samples was generated using the R package “corrplot.” To address a secondary research question on the impact of rock size on the community, continuous factor rock length was also included as a co‐variable to the PERMANOVA design.

The distance–decay relationship was explored in Stata‐14 using a generalized linear mixed model with a restricted maximum likelihood and the natural log of the Bray–Curtis similarities as outcome variable and the natural log of the distances between quadrats (+1) as fixed factor and the quadrat combinations as random factors. The model was superior to a linear regression (likelihood ratio test *p *<* *.001), and the standardized model residuals were normally distributed.

Different cluster approaches were compared in Primer‐7 (group‐average, single‐ and complete‐linkage, beta‐flexible), and the approach with the highest cophenetic correlation (0.89) was chosen. This was a group‐average hierarchical cluster approach based on the Bray–Curtis distance matrix and generated with a similarity profile (SIMPROF) test showing nodes with evidence of clustering at the 5% threshold.

Rarefaction curves and Chao‐1 richness estimates were calculated in Estimate‐S on the raw OTU data after exclusion of OTUs and samples as described above. Data were displayed using GraphPad Prism 6 (Graphpad Software, CA, USA).

To test for OTUs changing between distance classes, a negative binomial model was conducted in phyloseq on the variance stabilized OTU data implemented as described above.

Operational taxonomic unit data were log transformed in Stata‐14 and OTUs occurring at more than 0.1% in a sample were displayed in a Cytoscape network using the edge‐weighted spring‐embedded layout.

Secondary analyses addressed the question of the impact of rock type (crystal vs. matrix) and rock dimensions (mass, length, width, and thickness) upon the cyanobacterial community. A step‐wise distance‐based linear model and redundancy analysis (dbRDA) were performed in Primer‐7 with distance, rock type, and dimensions as predictors and using the Akaike information criterion (AIC) as selection criterion.

## 4 RESULTS

### 4.1 Processing the OTU data

The number of 16S rDNA sequences per sample varied between 8,094 and 68,775 sequences (median 17,483 sequences) with one sample (sample A5) only consisting of 4,530 sequences. This sample was excluded. From an initial 3,525 OTUs, 407 OTUs (11%) were excluded because they only occurred once in any of the 47 samples. A further 10 OTUs were excluded as they did not have a cyanobacteria taxonomic classification. Three of these 10 had no taxonomic match, three were of the order Rhodocyclales, one each from the orders Myxococcales, Burkholderiales, and Clostridiales and an OTU with a fungi classification of the order Glomerellales. This left 3,108 OTUs. There was no association between the number of sequences and OTUs in a sample (Spearman's rank correlation, *p *=* *.8). A second stage analysis on the correlation between Bray–Curtis distance matrices based on (1) standardized OTUs by relative abundance and square root transformed, (2) OTUs which were rarefied to 8,094 sequences and square root transformed, and (3) variance stabilized OTU data (McMurdie & Holmes, 2014) showed a high correlation (Spearman rank correlation coefficient >0.99) indicating that the effect of different standardization techniques upon the beta analysis was negligible in this study (data not shown). Furthermore, PERMANOVA analysis showed similar results for the three standardizing techniques (Table S1). Subsequent PERMANOVA analysis was conducted on standardized OTU data (1).

### 4.2 OTU richness

**Figure 2:**
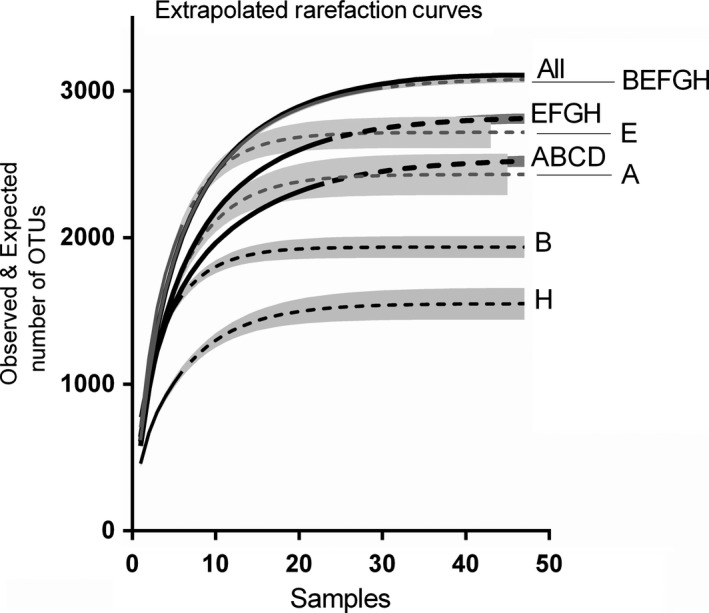
OTU rarefaction curves with observed (solid lines) and extrapolated (dashed lines) number of OTUs for 47 samples based on all samples, a combination of quadrats, or a selection of single quadrats. The shaded areas show the 95% confidence intervals

**Figure 3:**
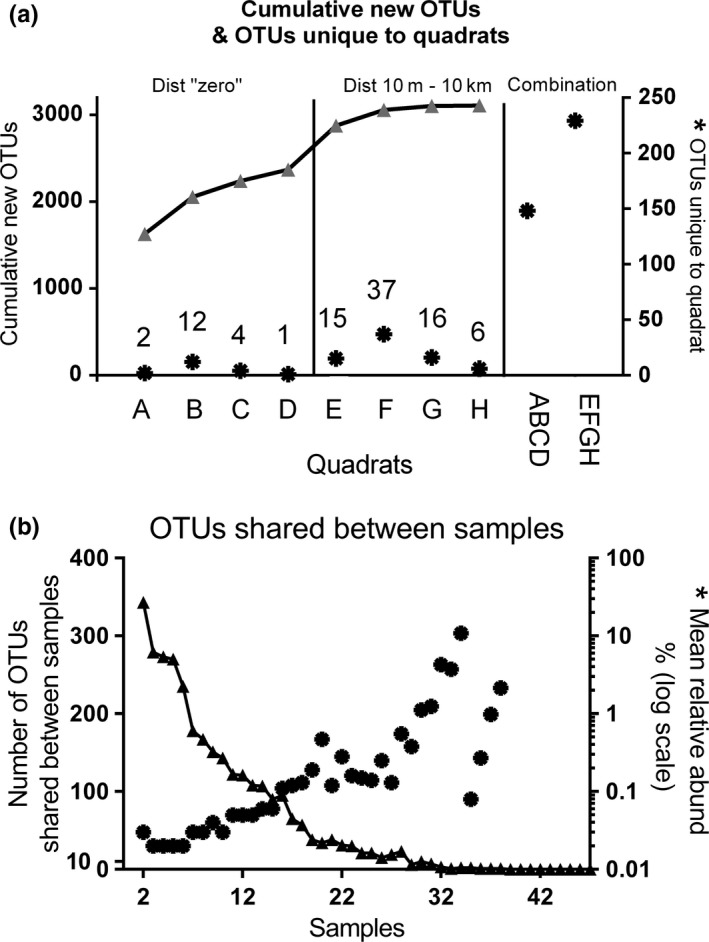
(a) Cumulative new OTUs starting at quadrat A (distance zero) and finishing at quadrat H (distance 10,000 m). The stars refer to the 2nd *y*‐axis on the right and show the number of OTUs unique for a quadrat or combination thereof, with the exact number shown above the symbols. (b) Number of OTUs shared between samples (triangles and left axis) and their average relative abundance in these samples (stars and 2nd *y*‐axis on the right)

With a total of 3,108 cyanobacterial OTUs detected in this study, an average of 580 OTUs were recovered from each rock, ranging from 106 to 1,081 OTUs (interquartile range 443–883 OTUs). A rarefaction analysis including all 47 samples showed a flattening of the curve corresponding with ~30 samples, suggesting that sampling additional rocks above this number only yielded a few additional OTUs (Figure 2). While curves also started to flatten for subsets of 23–24 samples, namely the combination of four quadrats ABCD at distance zero and EFGH at distances 10 to 10,000 m, neither of these extrapolated curves nor confidence intervals reached the total observed OTUs for all samples. This indicates that despite the flattening of the curves, additional OTUs were captured when rocks were sampled from additional locations, even if just 10 m away. With both quadrat combinations EFGH and ABCD consisting of a similar sample number, samples collected across most of the 10,000‐m transect (EFGH) detected 86% of OTUs (2,675 OTUs) while the same sampling effort at one location only (ABCD) recovered only 76% (2,370 OTUs) (Figure 3a). Similar results were obtained for Chao‐1 OTU estimates with EFGH covering 91% of all OTUs (2,823 OTUs) compared to 80% for ABCD (2,495 OTUs). No flattening of the rarefaction curve was evident considering only six rocks from one quadrat, indicating that six samples were not sufficient to capture the OTU richness at a location. OTU numbers varied widely between the quadrats, and the wide confidence intervals of extrapolated OTU numbers of some quadrats covered the observed OTUs of the corresponding quadrat combinations while others were considerably lower (Figure 2). Plotting the cumulative number of new OTUs from quadrat A to H revealed a steepening of the curve beyond ABCD which also indicated that sampling at new locations even if just 10 m away captured a considerably higher number of new OTUs compared to more intensive sampling at the same location (Figure 3a). The combination of EFGH revealed 229 unique OTUs, which compared to only 148 unique OTUs for ABCD (Figure 3a). However, a flattening of the curve beyond site F at 100 m indicated that the majority of OTUs were collected within the first 100 m of the 10‐km transect. Although the subsets described above consisting of four locations (ABCD and EFGH) did not reflect the entire community represented by all the quadrats, the subset BEFGH (all five locations, but only one quadrat from distance zero instead of four) did overlap the line produced by “All” quadrats (Figure 2).

Figure 3b shows that the majority of OTUs only occurred in a few samples. In fact, only one OTU was shared between 80% of samples (38/47) and it was identified as *Chroococcidiopsis* spp. Furthermore, only 133 OTUs (4.3%) occurred in more than half of the samples. Although they were only a small fraction of the total OTUs, the OTUs that were widespread among rocks (on more than half of the samples) were relatively abundant on the rocks. However, most OTUs were neither widespread nor abundant on rocks (Figure 3b).

**Figure 4:**
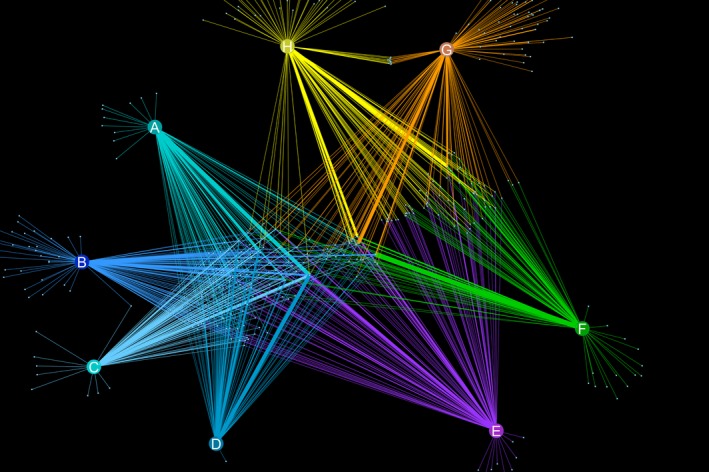
Cytoscape network showing all OTUs whose relative abundance was bigger than 0.1% in the corresponding sample. The thickness of the lines (edges) reflects the relative abundance of that OTU. The nodes mark the quadrats and OTUs

A network graph in Figure 4 shows OTUs occurring at more than 0.1% relative abundance distributed across sites. It again shows that those relatively rare OTUs that were shared among numerous samples occurred at higher relative abundance. The majority of OTUs from site H were shared with sites E, F, or G with considerably fewer OTUs from site H being shared exclusively with sites ABCD.

### 4.3 The cyanobacterial community composition

**Figure 5:**
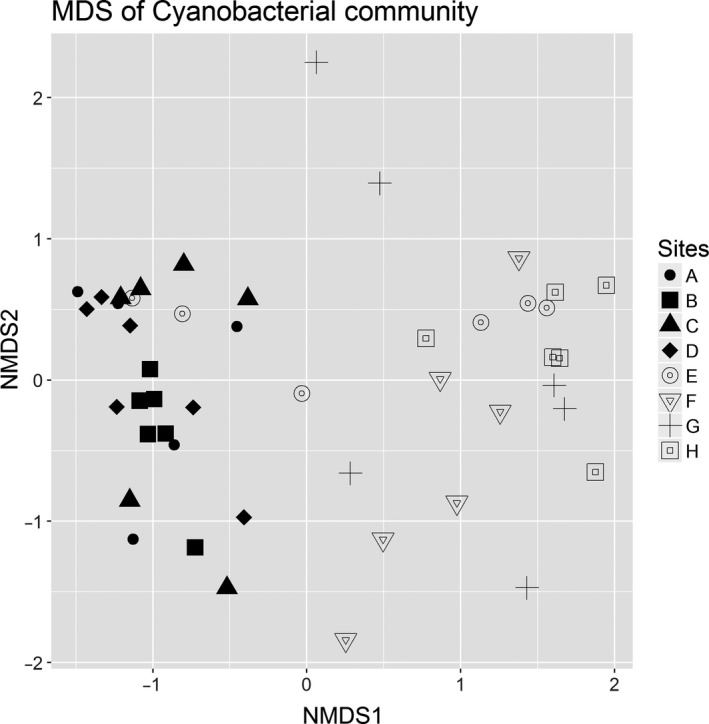
Relatedness of the cyanobacterial communities at sites as shown by a nonmetric multidimensional scaling (nMDS) of cyanobacterial communities sampled from distance 0 to distance 10,000 m. The nMDS was based on a Bray–Curtis distance matrix of the standardized and log transformed cyanobacterial OTU data. Each of the five subplots show the same nMDS but with samples corresponding to the distance class in the title of the subplot

**Figure 6:**
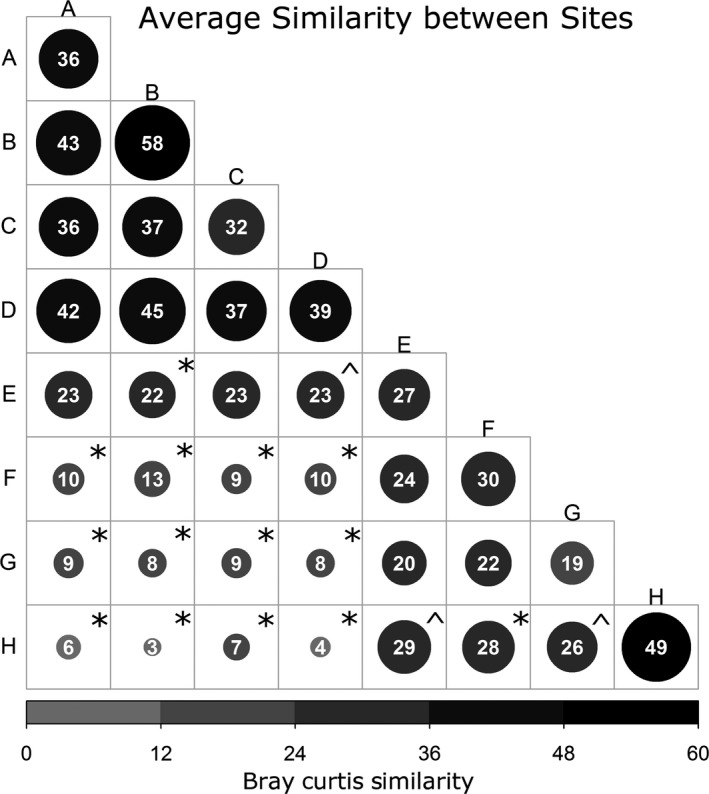
Heat‐map triangle showing the average Bray–Curtis similarities of the standardized and square root transformed cyanobacterial OTU data between sites. The circles on the diagonal show the within‐site similarities of the six rocks from the site. Larger and darker circles mark higher similarities within and between sites. Stars indicate significantly different communities based on pairwise PERMANOVA comparisons between sites at *p *<* *.01 and a caret for *p *<* *.05

Although there was no difference in the Shannon diversity of the cyanobacterial communities between sites (Kruskal‐Wallis test, *p *=* *.7), a nonmetric multidimensional scaling (nMDS) showed some clustering of the communities according to their distance class (Figure 5). In particular, the cyanobacteria collected from rocks at 10,000 m (Site H) clustered most tightly, while rocks at 10 m (site E) and 1,000 m (site G) varied most. This was also reflected by their average Bray–Curtis similarities, with sites B and H showing the highest within‐site similarities (58% and 49%) compared to sites G and E with the lowest within‐site similarities (19% and 27%; Figure 6). The clustering of communities according to their distance class was confirmed by the PERMANOVA analysis showing a significant difference in the communities between the distance classes (Table 1a). Based on the square root of the estimated component of variation, an average 47% of OTUs were dissimilar within a distance class while the composition of OTUs differed a further 38% between the distance classes (PERMANOVA *P *=* *.001) (Table 1a). A pairwise PERMANOVA comparison at site level showed no difference in the community composition between sites A to D at distance zero (Figure 6), but the combined community at distance zero (A to D) was significantly different from all other distances (pairwise PERMANOVA at distance‐level *p *<* *.004 for all comparisons) (Table 1b). Site H at 10,000 m significantly differed from all other sites including F (100 m) and G (1,000 m) (Figure 6). A distance‐based test for homogeneity of multivariate dispersions (PermDISP) showed some evidence that the variance of the community data differed between the distance groups (*p *=* *.016), in particular between the distance class 10,000 and 1,000 or 100 m and between 0 and 1,000 m (.01 < *p *<* *.05). Thus, the differences in dispersion of the communities at sites F (100 m) and H (10,000 m) contributed to the PERMANOVA test result indicating a significant difference in the cyanobacterial community between these two sites.

**Table 1:** PERMANOVA Pseudo‐*F* statistic to test for differences in the cyanobacterial communities between distances

a)					
Factor	*df*	Pseudo‐*F*	Sqrt‐CV	*p* value	Perms
Distance	4	6.2	37.5	.001	999
Residual	42		47.1		

b)			
Distance levels	*t* test	*p* value	Perms
0–10	2.2	.003*	999
0–100	3.1	.001*	996
0–1,000	2.8	.001*	999
0–10,000	3.9	.001*	999
10–100	1.2	.166	410
10–1,000	1.1	.195	411
10–10,000	1.6	.044^	422
100–1,000	1.1	.153	404
100–10,000	1.8	.009*	402
1,000–10,000	1.5	.025^	396

The analysis is based on a Bray–Curtis distance matrix of square root transformed and standardized OTU abundances. (a) Main test with fixed factor distance and (b) pairwise testing for differences in the communities between distance levels.

*df*, degrees of freedom; Sqrt‐CV, the square root of the estimated component of variation. This indicates the size of the effect in the unit of the Bray–Curtis dissimilarity matrix, that is, % of dissimilar OTUs; Perms, number of permutations.

Stars (*) indicate *p *<* *.01 and a caret (^) indicates *p *<* *.05.

**Figure 7:**
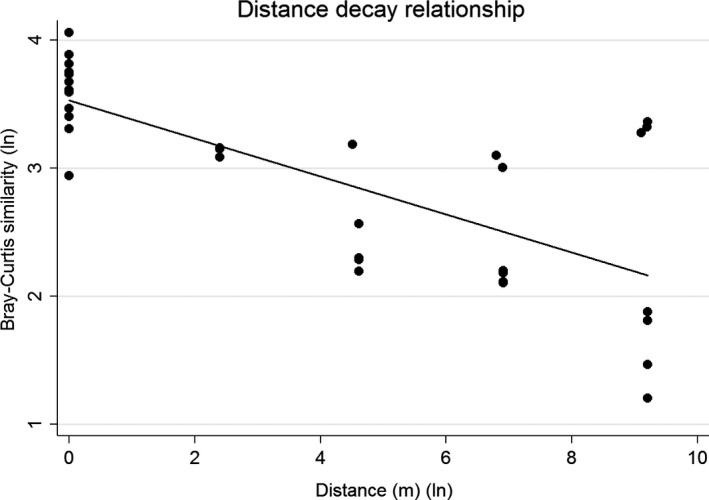
A scatter plot with the natural log transformed Bray–Curtis similarities between the cyanobacterial communities over the natural log transformed distance (+1) between the quadrats. The line shows the linear fit with a negative slope significantly different from zero (*p* < .001)

A distance–decay analysis showed that the Bray–Curtis similarities between the cyanobacterial communities significantly decreased with increasing distance between the quadrats (Figure 7). For a 10% increase in the distance, there was an average 1.8% decrease in the Bray–Curtis similarity (*p* < .001).

Twenty‐two genera and 10 families of cyanobacteria were found in this study, and Table 2 lists the most commonly found taxa. With increasing distance, there was a shift in the distribution with a larger relative abundance of Phormidiaceaea at the more distant sites and less Nostocaceae. This was also supported by a negative binomial model on the variance stabilized OTU data (McMurdie & Holmes, 2014). Six of the 10 OTUs which increased most at the more distant sites belonged to the Phormidiaceaea family, with the four remaining OTUs being Spirulinaceae, Chamaesiphonaceae, and Chroococcidiopsis. The top 10 OTUs which decreased most with increasing distance belonged to the Chroococcidiopsis and Nostocaceae families (*p* values adjusted for multiple testing *p *<* *1 × 10^−6^ for all).

**Table 2:** A list of the top 20 (average relative abundance of sequence counts) taxa for combined sites ABCD ranked from most to least abundant

Taxa	Average sequence relative abundance				
	ABCD	E	F	G	H
*Nostoc ellipsosporum*	7,930	3,912	7,599	3,173	1,375
*Nodularia sphaerocarpa*	7,245	4,152	4	293	75
*Phormidium phormidiaceae cyanobacterium*	2,161	750	0	24	58
*Phormidium pristleyi*	1,691	3,188	3,515	3,993	3,747
*Maritimimonas* spp.	1,593	926	37	2,566	11
*Planktothricoides raciborskii*	1,382	540	1	47	32
*Microcoleus vaginatus*	855	3,098	2,131	1,000	2,305
*Leptolyngbya antarctica*	240	209	446	363	314
*Fischerella muscicola*	185	88	414	137	20
*Microcoleus chthonoplastes*	68	203	77	159	40
*Oscillatoria spongeliae*	55	779	505	237	778
*Xenococcus* spp.	51	345	395	464	141
*Acidovorax* spp.	45	3	14	4	0
*Thermosynechococcus elongatus*	40	92	36	156	60
*Oscillatoria* spp.	37	23	63	36	5
*Cycloclasticus* spp.	17	91	49	21	43
*Anabaena cylindrica*	16	6	8	7	4
*Leptolyngbya laminosa*	15	12	0	0	6
*Chondromyces lanuginosus*	8	91	47	8	58
*Spirulina* spp.	3	3	0	1	0
*Nostoc piscinale*	2	52	102	105	65
*Lyngbya polychroa*	1	0	13	3	0
*Gloeotrichia* spp.	0	52	7	6	12
*Geitlerinema* spp.	1	28	18	3	14
*Nostoc* spp.	0	27	17	16	9

The last five taxa listed were included because they were in the top 20 most abundant taxa for sites E, F, G, or H.

### 4.4 The cyanobacterial community and rock characteristics

Table 3 shows the range of rock dimensions and type per distance class. There was no difference in the rock types between the distance classes (Fisher's exact test, *p *=* *.13). However, rocks collected at distance zero were significantly smaller (Table 3) compared to all other sites (Dunn's pairwise comparison *p *<* *.05 (FDR adjusted for multiple testing). No difference in the number of OTUs, OTU richness, nor Shannon diversity was found between different rock types, rock weight, or linear dimensions (Mann‐Whitney tests or Spearman's rank correlations, *p* value ranged from .09 to .9 for all comparisons). A negative binomial model (McMurdie & Holmes, 2014) did not return any OTUs which significantly differed between rock characteristics. In order to test whether rock characteristics had an effect on the community structure, a distance‐based linear model and redundancy analysis (dbRDA) was conducted with factors distance class, rock size (weight and size dimensions), and rock type (crystal vs. matrix). This analysis showed that distance explained 13.3% (*p *=* *.001) of the variation in the bacterial community. An additional 5.0% of the variance was explained by rock length (*p *=* *.011) and 4.6% by rock type (*p *=* *.011) while rock weight did not contribute to the model (Fig. S1). To assess whether rock length explained some of the differences in the bacterial community observed between the different distance classes, rock length was added as a continuous covariable to the PERMANOVA analysis with distance as a fixed factor. Distance remained the largest source of variation in the bacterial community data with a square root of the component of variation of 39% (*p *=* *.001) compared to 13% for rock length (*p *=* *.001) (residual 46%). There was a weak interaction effect between distance and rock length (*p *=* *.06) indicating that the impact of distance upon the community slightly differed based on rock length. A pairwise PERMANOVA comparison of communities between distances with length as cofactor returned a more significant *t*‐statistic for pairs with rocks at distance 10 m with a *p *<* *.01 comparing 10 m with 10,000 m and *p *=* *.032 comparing 10 m with 100 m. Rocks at distance 10 m showed the largest variation in length including the longest rocks. Accounting for length also lowered the *p* value to below .01 for the comparison of communities between 1,000 and 10,000 m.

**Table 3:** Rock characteristics per distance group

Distance (m) (sites)	Crystal type (%)	Mass (g)	Length (mm)	Width (mm)	Thickness (mm)
0 (A–D)	71	10.4 (4.8–33.1)	26.4 (12.1–40.8)	20.0 (6.7–33.4)	16.7 (6.0–24.4)
10 (E)	50	108.4 (9.3–1,185)	62.7 (24.7–127)	43.5 (17.1–110)	18.8 (12.9–54.7)
100 (F)	83	141.9 (9.7–631)	63.9 (30.0–113)	38.2 (20.6–92.7)	28.2 (13.6–47.2)
1,000 (G)	67	100.1 (14.6–723)	47.5 (23.5–121)	32.4 (21.3–61.0)	27.3 (16.3–37.0)
10,000 (H)	17	65.8 (15.9–153)	50.9 (38.7–64.0)	38.1 (27.1–61.6)	21.7 (9.3–33.2)

Numbers are median rock characteristics with the range in brackets. Crystal type is expressed as the percentage of the rocks that were crystal type, with the remainder being matrix type.

Overall, rock length accounted for some of the differences in the cyanobacterial communities, and by accounting for these differences, the size of the effect due to distance increased even more for some pairwise comparisons.

## 5 DISCUSSION

At a landscape scale, large enough for climatic variability, hypolithic cyanobacterial abundance (as measured by percentage of colonization of available rocks) and diversity increase with increasing moisture in the environment (Cowan et al., 2011; Heckman et al., 2006; Pointing, 2016; Warren‐Rhodes et al., 2006, 2013). At a more local scale, the patchy distribution of hypolithic communities has been attributed to a combination of topographic characteristics and dispersal characteristics such that the presence of a colonized stone facilitates the colonization of near‐by stones (Pointing, 2016; Warren‐Rhodes et al., 2007). Rates of colonization are not well known, but are likely to be related to water availability and, in particular, runoff. The uncertainties associated with runoff, coupled with the possibility of noncontinuous patches of appropriate rocks, give rise to the possibility that distance, even in an environment with relatively homogeneous climate and topography, may be an effective barrier to dispersal and species distributions. Thus, a distance–decay effect is likely to be important over some geographic scales (Lacap et al., 2011).

In this study, the species richness did not increase substantially over the 10‐km transect as was hypothesized on the basis of the original study from this semi‐arid region (Tracy et al., 2010). However, as shown by the cumulative plot of OTUs with increasing distance (Figure 3a), substantial numbers of additional OTUs were recorded at each site up to the first 100 m (ABCDEF; Figure 3a). The cumulative plot levels‐off after 100 m and there were only 45 additional OTUs recorded at 1,000 m (G) (1.5% of 3,102 OTUs cumulated to quadrat G), and six additional OTUs at the 10 km site (H) (0.2% of 3,108 OTUs cumulated to H). Thus, to sample the bulk of the community composition of a region requires a sampling effort on the scale of ~100 m, but additional diversity may be added with the inclusion of more distant sites.

Our results indicate that distance can be a determinant of cyanobacterial community composition at a scale as small as 10 m as evidenced by the cyanobacterial community at distance zero being significantly different from that at 10 m (Figure 6). However, this distance effect also depended on the variability of inter‐rock communities at a site, as illustrated by the cyanobacterial communities at the sites at 10 m (E) and 1 km (G) which had low within‐site similarity and which did not differ from each other nor those at 100 m (F) (Figure 6). The site at 10 m showed large variations in rock lengths and once differences in the communities due to differing rock lengths were accounted for, the communities at 10 m also significantly differed from most other distances. Overall, our results indicate that, over the 10‐km transect, distance was a significant factor that is confounded by inter‐rock differences at some locations (such as the sample at 10 m, and to a lesser extent at 1 km).

Distance is an important factor in determining community composition (Martiny et al., 2011), but we cannot distinguish between the effects of distance *per se* and inconspicuous, subtle environmental factors that vary with distance and therefore confound the effect of distance itself. However, the habitat at distances 0, 10, and 100 m was extremely homogeneous (extremely flat with no discernable differences in vegetation or soil), and the fact that there were such pronounced differences in community composition among these close distances suggests that the most parsimonious explanation is that distance itself is a barrier to dispersal. Nevertheless, an in‐depth examination of soil chemistry would be a valuable contribution to future studies.

The number of OTUs per rock did not differ among the locations, but, to some extent, the composition did. At each distance, the community had OTUs not found elsewhere. However, the community at the most distant location was not radically different from the other sites. These data allow us to address the question “How many rocks need to be sampled to have a fairly complete picture of the community composition at a given location?” The answer to this question depends on distance (i.e., the size of the “location”), but at the scale of 10 km, ~30 samples, collected along the 10 km transect, are required. This is based on inspection of Figure 2 which asymptotes at about that number of samples when six rocks were collected from each of five sites along the 10‐km transect. The sample of 23 rocks from distance zero retrieved only ~76% of all OTUs found from the total sample of 47 rocks. The same sampling effort (23 rocks) from the quadrats from 10 m to 10 km (EFGH) would have recovered more, that is, 86% of all OTUs, and sampling 30 rocks across the entire transect (BEFGH) would have resulted in a recovery of 97% of all OTUs at a lower effort and less expense. However, the intensive sampling effort at distance zero (ABCD) nevertheless resulted in unique OTUs that would not have been found if only one of the four quadrats had been sampled (87–133 OTUs or 2.8%–4.3%, depending on which of the four quadrats was selected). It is clear that there is high cyanobacterial diversity in this environment, and as the cost of next generation sequencing goes down, the ability to examine more rocks will increase. In the meantime, these results give an indication of the sample sizes required to adequately describe the cyanobacterial community on the local scale.

Although the objectives of this study did not include an investigation of colonization, the results can be viewed in relation to models that attempt to describe how cyanobacteria move among rocks and colonize new rocks with the appropriate characteristics and microhabitats (Tracy et al., 2010). In a study of the bacterial communities in the Namib Desert, Makhalanyane, Valverde, Lacap et al. (2013) concluded that although the cyanobacterial community under rocks was distinct from the surrounding soil bacterial community, there was nevertheless substantial overlap between the two microhabitats, with 88% of the taxonomic units in the hypolithic environment also being found in the soil. Thus, these authors conclude that the hypolithic community is recruited as a selective subset of the soil microbiota. This model of recruitment is at odds with a colonization model in which hypolithic cyanobacteria are viewed as specialists that must disperse from one rock microhabitat to another, presumably facilitated by water runoff (Pointing, 2016; Warren‐Rhodes et al., 2007). Given the photosensitivity of hypolithic cyanobacteria (Tracy et al., 2010), it seems unlikely that hypoliths could be metabolically active either on the surface of the soil where the photosynthetically active radiation would be too intense or under the relatively opaque soil, where the radiation would not penetrate. A nanohabitat at a soil depth appropriate for photosynthesis to occur would seem to be impossible, or at least impossibly unstable and subject to the slightest disturbance. A more parsimonious explanation for the results of Makhalanyane, Valverde, Lacap et al. (2013) would be that the hypolithic cyanobacterial DNA detected in the soil was of dormant (Lacap et al., 2011) or relic (Carini et al., 2016) cyanobacteria rather than an active component of the soil microbiota. Thus, from an ecological perspective, the detection of dormant cyanobacteria in soil would be an indicator of potential colonizers in transit (Pointing, 2016; Warren‐Rhodes et al., 2007) rather than being part of the functional soil community. These two models, in which hypolithic cyanobacteria in soil represent either active, growing bacteria that could spread intrinsically, or dormant particles dependent on the vagaries of runoff, would result in very different patterns of dispersal and recruitment. Alternatively, the detection of relic cyanobacterial DNA in soil would simply reflect the long‐term persistence of extracellular DNA from dead microorganisms (Carini et al., 2016). Surveys using RNA‐based approaches are required to differentiate among these possibilities (Lacap et al., 2011).

Within a quadrat (comparing the six replicate rocks), the cyanobacterial communities in most quadrats had high similarity, and this pattern extended to the four adjacent quadrats at distance zero, which is consistent with the observations of Wong et al. (2010). However, at a distance of 10 m, there was a distance effect compared to the cyanobacterial communities at distance zero, indicating that proximity influences community composition. The community at distance zero was also significantly different from that at all of the more distant locations (100 m, 1 km, and 10 km) (Figure 6). These patterns of distribution are more easily explained under a colonization model in which hypolithic cyanobacteria disperse and colonize from rock to rock (with a possible dormant stage between rocks) (Pointing, 2016; Warren‐Rhodes et al., 2007) rather than them being active components of the soil microbiota that are recruited to form hypolithic communities (Makhalanyane, Valverde, Lacap et al. 2013).

Although much has been learned about hypolithic cyanobacterial communities in recent years (Pointing, 2016), there are still many unanswered questions. These include the relationships between soil microbiota and hypolithic microhabitats, the dispersal and colonization mechanisms involved in inoculating available rocks, rates of colonization and the factors that determine it, and the interactions among species that have colonized a rock. At the landscape scale, further work is required to document the presumed functional roles of cyanobacterial communities in arid and semi‐arid regions.

## CONFLICT OF INTEREST

None declared.

## AUTHOR CONTRIBUTIONS

K.C. collected the samples in the field, K.C. and K.G. extracted the DNA, M.K. analyzed the data, K.C., with assistance from M.K. and K.G., wrote and revised the manuscript.

## DATA ACCESSIBILITY

Sequences from this study have been deposited in the NCBI Sequence Read Archive under accessions: STUDY: PRJNA377425 (SRP102841), SAMPLE: 082614KG359F (SRS2091045), EXPERIMENT: A1_H6 (SRX2694711), RUN: SRA sub 082614KG359F‐pr.fasta.otus.fa (SRR5399968). Available via SRA RunSelector: https://www.ncbi.nlm.nih.gov/Traces/study/?acc=SRP102841.
