# Supplementary material for: Spatial patterns of hypolithic cyanobacterial diversity in Northern Australia
Source: Ecol Evol. 2017 Jul 31;7(17):7023–33. doi: 10.1002/ece3.3248 (PMC5587464; doi:10.1002/ece3.3248)

**Supporting information**

**Supplementary Table 1**

PERMANOVA Pseudo-F statistic to test for differences in the cyanobacterial communities between distances. The analysis is based on a Bray Curtis distance matrix:

**(a)** standardized OTUs by relative abundance of square root transformed OTU counts,

**(b)** OTUs which were rarefied to 8,094 sequences and square root transformed,

**(c)** variance stabilized OTU data (McMurdie *et al*. 2014).

| Transformation of OTU counts | Factor | df | Pseudo-F | Sqrt-CV | *P* value | Perms |
| --- | --- | --- | --- | --- | --- | --- |
| (a) | Distance | 4 | 6.2 | 37.5 | 0.001 | 999 |
|  | Residual | 42 |  | 47.1 |  |  |
| (b) | Distance | 4 | 5.9 | 37.3 | 0.001 | 997 |
|  | Residual | 42 |  | 48.2 |  |  |
| (c) | Distance | 4 | 6.7 | 37.8 | 0.001 | 998 |
|  | Residual | 42 |  | 45.0 |  |  |

Abbreviation: df, degrees of freedom; Sqrt-CV, the square root of the estimated component of variation. This indicates the size of the effect in the unit of the Bray Curtis dissimilarity matrix i.e. % of dissimilar OTUs; Perms, number of permutations.

**Supplementary Figures**

**Fig. S1** A dbRDA on the cyanobacterial community with predictors distance and rock characteristics. The final model included distance, rock type (crystal, c versus matrix, m) and rock length.


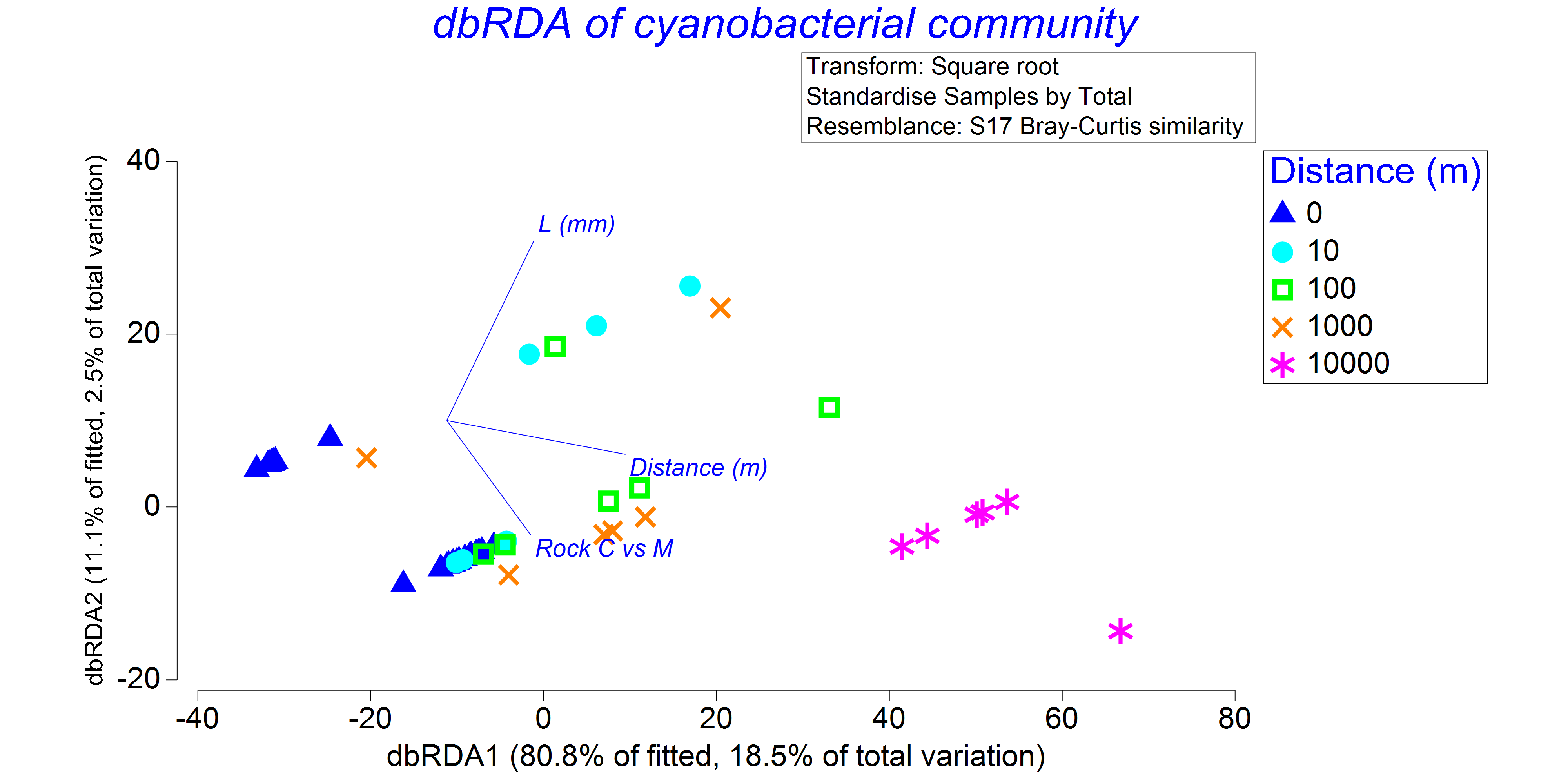

Supplement: Supplementary file 1 [file ECE3-7-7023-s001.docx]
